# Sugar-Triggered Burst Drug Releasing Poly-Lactic Acid (PLA) Microneedles and Its Fabrication Based on Solvent-Casting Approach

**DOI:** 10.3390/pharmaceutics14091758

**Published:** 2022-08-23

**Authors:** Seongsu Kang, Ji Eun Song, Seung-Hyun Jun, Sun-Gyoo Park, Nae-Gyu Kang

**Affiliations:** LG Household and Health Care R&D Center, Seoul 07795, Korea

**Keywords:** microneedle, poly-lactic acid (PLA), solid microneedle, transdermal delivery, solvent-casting

## Abstract

Microneedles have emerged as a novel transdermal delivery tool that enables the delivery of various products such as drugs, vaccines, or cosmetic ingredients. Although the demand for solid microneedles composed of biocompatible polymer is increasing, the manufacture of microneedles using poly-lactic acid (PLA) with rapid drug-releasing is yet to be established and the process is still in its infancy. Here, we propose a novel strategy for the fabrication of PLA solid microneedles which enable a drug to be burst-released based on a solvent-casting process. This approach offers extreme simplicity, broad geometric capability, cost-effectiveness, and scalability based on high fidelity-replicas. It was verified that microneedles of various heights (250–500 μm) could be fabricated with appropriate mechanical strength to penetrate the stratum corneum layer of skin. By adding sugar in the composition of PLA microneedle, it was observed that both hydrophilic and hydrophobic drugs can be rapidly released within 30 min. Our burst drug-releasing PLA microneedle having both characteristics of solid microneedle and soluble microneedle and its fabrication approach based on solvent-casting will contribute to getting microneedle technology close to commercialization and beyond existing technical limitations.

## 1. Introduction

The skin is the largest and most accessible organ of the body, with a surface area of 2 m^2^ in adults. Various types of exogenous molecules can be delivered into the body through the skin using what is generally termed a transdermal delivery system (TDS). The transdermal delivery of exogenous molecules is strictly limited by the target molecule’s size (<500 Da) and the balance between hydrophilicity and hydrophobicity (log P = 1–3) [1,2]. To overcome this drawback, special methodologies are required that can achieve efficient transdermal delivery.

Among the various methods used for transdermal delivery (such as sonophoresis, electrophoresis, or iontophoresis) microneedles have received great attention. These novel systems comprise miniaturized needles (length = 150–1500 μm) with diverse geometries. They have the potential to overcome the limitations of conventional approaches. Microneedles enable the delivery not only of small molecules, but also of macromolecules such as antibodies. Hydrophilic or hydrophobic drugs can also be delivered though the micro-holes that these microneedles physically create in the stratum corneum [3]. Such penetration generally does not trigger pain or severe bleeding, thereby enabling self-application with high patient compliance. These advantages have led to the use of microneedles in a variety of applications, such as pharmaceuticals, medical devise sensors, and cosmetics [2]. For this reason, some microneedle-based products are commercially available under various claims, and few microneedle-based drugs are also under clinical trials [4]. It is expected that the microneedle-based drug delivery system market will approach $1.2 billion with CAGR (compound annual growth rate) of 6.6% by 2030 [5]. Microneedles are generally classified as either (a) solid microneedles that are used to form micro-holes in the skin as pretreatment (sometimes with a drug coated substrate), (b) hollow microneedles for the delivery of liquid flowing continuously through fine channels, or (c) soluble dissolving microneedles that comprise a solidified drug substrate [6,7]. Solid microneedles have been made using materials such as metal, silicon, and various polymers so as to achieve sufficient mechanical strength. However, some materials such as silicon or metal can constitute a safety risk to user based on insufficient evidence against their biocompatibility or a concern regarding granulomas when they remain in the body [8,9]. In recent years, various biodegradable and biocompatible polymers such as polystyrene, polyvinyl pyrrolidone, polycarbonate, poly-lactic acid (PLA), and poly(lactic-co-glycolic acid) (PLGA) [10] have been used to fabricate solid microneedles. Among these materials, PLA has been widely used in biomedical engineering applications across a diverse range of formulations, including films, sheets, microspheres, implants, and plates [11,12].

However, solid microneedles require an external drug supplier or coating process, unlike soluble or hollow-type microneedles. For this reason, a diverse range of approaches have been studied, including robust and efficient coating technologies, combining external drug reservoirs, and loading target drugs into the solid microneedle’s structure itself to ensure its sustained release. It has been demonstrated that drugs can be loaded into microneedles comprising PLGA or PLA matrixes, with such systems demonstrating a sustained release of the target drug [13,14,15,16]. This approach generally requires more than 24 h or even 20 d for the effective delivery of the target drug [15,16]. Though the delayed sustained-release is suitable for hormone-type drugs, it could limit the patient compliance due to a long time of application, especially for a prompt vaccination or a cosmetic use. In other words, a more rapid drug-releasing microneedle a with shorter application time, along with a fabrication approach which is technically simple and reliable, is required.

Here, we introduce bursting, drug-releasing PLA microneedles and detail their fabrication using a solvent-casting method that is compatible with mass production. Briefly, we dissolved an appropriate ratio of amorphous poly(D, L-lactide), the target drug, and sugar in dimethyl sulfoxide (DMSO), before pouring this into a polydimetylsiloxane (PDMS) microneedle mold. We analyzed the drug release profiles according to the composition and explored the temporal characteristics of drug release. We also examined our microneedles’ practical transdermal delivery for both hydrophilic and hydrophobic drugs. Bursting, drug-releasing PLA microneedles have the potential to greatly improve patient compliance and solve current issues regarding storage and packaging. Furthermore, by developing the abovementioned fabrication approach, we hope to contribute not only to cosmetic or pharmaceutic industries, but also to further scientific discoveries and applications regarding microneedles.

## 2. Materials and Methods

### 2.1. Fabrication of Microneedles

Resomer^®^ R Poly(D,L-lactide) series (203R, 205R, 207R), which have been used in medical devices and tissue engineering due to their biodegradable, biocompatible, and bioresorbable characteristics, were used in this study [17]. Three types of Resomer^®^ R, 203R, 205R, and 207R, were prepared. According to manufacturers, Resomer^®^ 203R, 205R, and 207R have the range of inherent viscosity (IV) of 0.25–0.35, 0.55–0.75, and 1.3–1.7, respectively. Before the solvent-casting process, PLA was degassed under vacuum for 1 h to eliminate water. The dried PLA was mixed with solvents in various concentrations and mixed gently until complete dissolution. The viscous PLA solution was centrifuged for 2 min with 2000 rpm to eliminate bubbles and to avoid air entrapment during casting process. (Centrifugal degassing procedure) The solution was poured into a polydimethylsiloxane (PDMS) mold having diverse pyramid-structure geometries with heights of 250, 300, 350, and 500 μm and an aspect ratio of 2. The center-to-center distance of each microneedle structure is 400, 600, 600, and 1000 μm for microneedle arrays of 250, 300, 350, and 500 μm height.

The PDMS prepolymer solution (Sylgard 184) mixed with curing agent was poured over a metal master mold (LG Electronics, Pyeongtaek, Korea) and cured overnight in an oven at 50 °C. The inverse replica of the PDMS mold was removed from the metal master mold, and used as the negative-mold for the PLA microneedles. The prepared PLA solution was cast on the PDMS negative-mold, vacuumed for 30 min, and dried overnight at 50 °C.

After overnight drying, the “dried” PLA structure was removed from the PDMS mold. The PLA microneedle arrays were washed using distilled water, and dried under ambient conditions at room temperature.

### 2.2. Characterization of the Microneedles

The mechanical property and strength of the microneedles was measured using a texture analyzer TA. XTplusC (Stable Micro System, Godalming, UK). Briefly, the microneedle arrays were attached to the bottom of a mechanical sensor, and the sensor was moved axially at the test speed of 0.1 mm/s with trigger force of 10 g. The single microneedle structure was measured at the test speed of 1.2 mm/min with a trigger force of 0.003 N. The microneedle structure was observed under an optical microscope (Leica DM 1000, Leica, Solms, Germany) and a scanning electron microscope (SEM, S-4800, Hitachi, Tokyo, Japan). The measured mechanical strength of the microneedle array was 200 μm of strain.

### 2.3. Skin Penetration Test

To investigate whether PLA microneedles having a height of 250 μm can be applied to human skin multiple times, the microneedle array was applied to a human volunteer (one of the authors, male, 29 years old) under gentle thumb pressure. A 12.5% solution so gardenia blue color (extracted from the rubiaceous plant gardenia fruit) was poured on the skin for 15 min, and then washed out. The stained pores of the stratum corneum were observed under an optical microscope. This study was approved by the Ethics Committee of the LG H&H Institutional Review Board (LGHH-20200709-AA-03). Prior to participation in the study, human test subject was informed of possible side effects and consented to participate in the study.

### 2.4. Transdermal Delivery In Vitro Using Franz-Type Diffusion Cells

A porcine skin membrane having the dimensions 2.5 × 2.5 cm was purchased (Micropig Franz Cell Membrane, Apures, Pyeongtaek-si, Korea). Before the experiment, the skin membrane was immersed in PBS (Phosphate buffered saline pH 7.4, Gibco, Waltham, MA, USA) and incubated for 2 h. In the following experiment, the surface of the stratum corneum was wiped with a paper tissue to get rid of the extra PBS. For the application of microneedles, the array was inserted into the skin under gentle thumb pressure. The reservoir was filled with PBS. The diffusion cells were tightly assembled with clamps and placed in an incubator at 37 °C and 50% relative humidity (RH). To quantify the target molecule in the stratum corneum, epidermis, and dermis, respectively, the porcine skin was tape-stripped three times and heated at 90 °C for 50 s. After separation of the epidermis and dermis, each compartment was homogenized with PBS and centrifuged. The clear supernatants were recovered and analyzed using photoluminescence spectroscopy (Varioskan™ LUX, Thermofisher, Waltham, MA, USA) to quantify the FITC and an Ascorbic Acid Assay Kit (L-Ascorbate, K-ASCO, Megazyme, Bray, UK) to quantify the vitamin C. The amount of retinol was analyzed by HPLC. Detailed analysis followed the previous literature [18]. 

For the transdermal delivery of ascorbic acid and FITC, ascorbic acid and FITC were prepared with concentration of 25% and 50,000 ng/mL, respectively. A 25% solution of vitamin C was titrated to pH 3.2 with KOH.

## 3. Results and Discussion

### 3.1. Fabrication of the Microneedles

Since the 1970s, PLA has been approved by the US Food and Drug Administration (FDA) for food and pharmaceutical applications with renewability, biocompatibility, and process capability as prime benefits [11]. It is known that PLA degrades inside the body by hydrolysis of the ester-bond back bone and enzymatic process, and its degradation in the human body and later excretion was also verified [19]. In particular, fabrication using PLA enables design of complex and sophisticated items such as tissue scaffolds. Recently PLA has also been used for the fabrication of microneedles based mainly on thermoforming [20]. However, the micro molding of PLA based on thermoforming shows some disadvantages such as requiring the heating system to maintain a temperature above 160 °C, limited resolution, and perturbation of the quality and molding efficiency depending on the operation conditions. It also produces excess polymer waste due to the nature of the process. 

**Figure 1:**
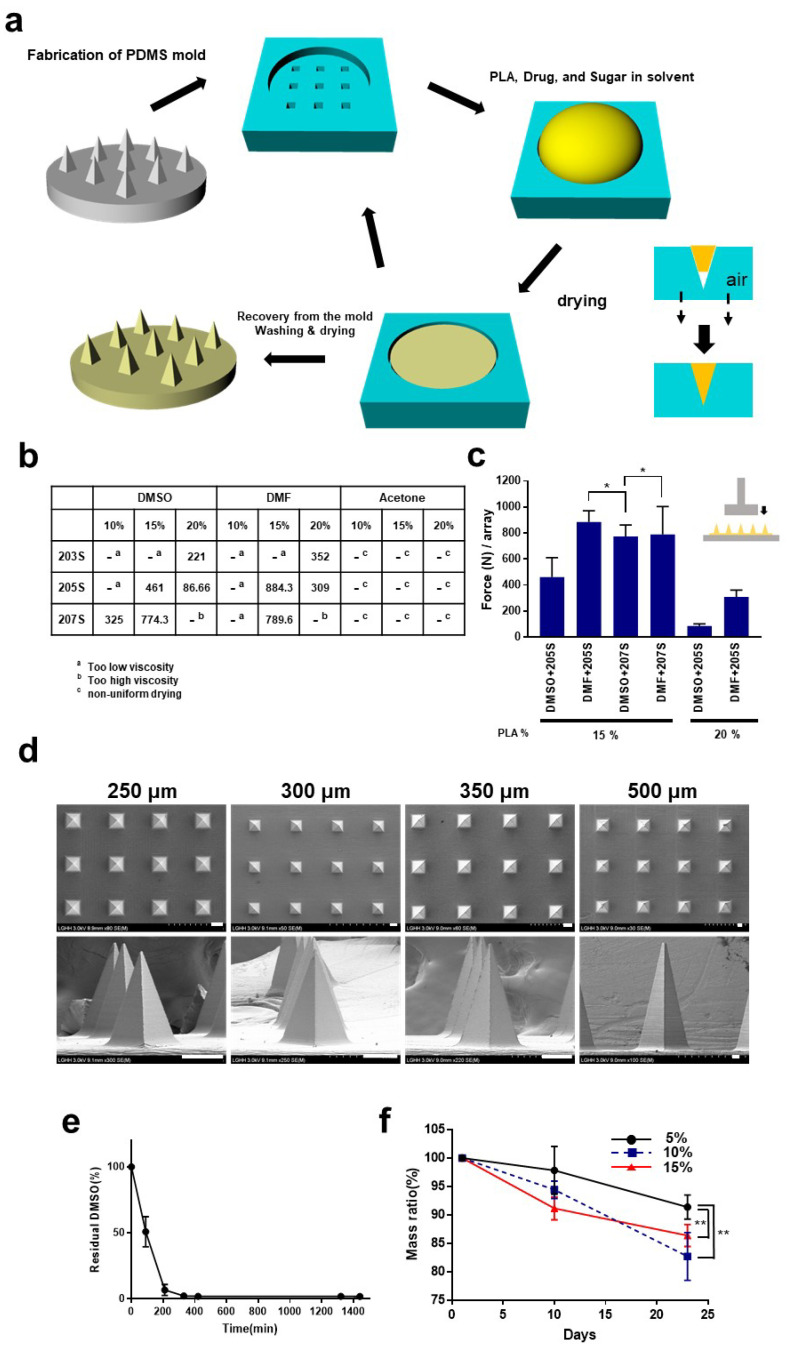
Fabrication and characterization of PLA microneedle based on solvent-casting. (**a**) Schematic diagram of fabrication of PLA microneedle. (**b**) Mechanical strength of microneedle of 300 μm height under various conditions (solvent, PLA molecular weight, weight % in solution). In the force-distance curve, the force at 200 μm was measured and noted in the table. Unit; Force (N)/array. (**c**) The leading candidates of fabrication conditions. The data originates from (**b**). (**d**) Scanning electron microscopy (SEM) image for microneedle array fabricated by Resomer 207S in DMSO with 15%. Scale bar (white) shows 100 μm. (**e**) Residual of DMSO during drying. The residual DMSO percentage was calculated in the following manner: 100 × [DMSO weight theoretically calculated (0 min) − (Weight at 0 min − Weight at each time point)]/DMSO weight theoretically calculated (0 min). The solution of Resomer 207S in DMSO with 15% was prepared in PDMS mold and dried. (**f**) Biodegradability of PLA microneedle. The PLA microneedle (Resomer 207S in DMSO with 15%) was incubated in proteinase K–contained PBS at 37 °C. Before each measurement of weight, the microneedle array was washed using distilled water and dried to eliminate the water. ** significantly different (*p* < 0.05); * significantly not different (*p* > 0.05) Student’s *t* test was performed.

First, we developed the fabrication process for PLA microneedle using solvent casting under low temperature which can be further compatible with the fabrication of the burst drug-releasing microneedle. A schematic diagram of the fabrication process is shown in Figure 1a. As described there, the PDMS was used as a negative mold for the PLA microneedles. Our fabrication method is based on micro molding which has been widely used for fabrication of microneedles in the last few decades [21]. Previous research showed that some organic solvents cause deformation or shrinkage of the PDMS, so an appropriate solvent should be considered for applications in which PDMS is utilized (such as microfluidics, chemical reactors, and solvent-casting-based scaffolds [22]. In addition, whether the solvent is bio-friendly and bioavailable without causing any harm to human was also considered for our study, even if excess solvent remains after drying. Based on these criteria, we chose three solvents, acetone, dimethylformamide (DMF), and, dimethyl sulfoxide (DMSO). Previous pioneering research, which examined the compatibility of various organic solvents with PDMS, also verified that the three solvents mentioned above do not show significant shrink or deformation of PDMS when they were applied on it [22]. 

We fabricated microneedles 300 μm high by varying the kinds of solvents, the molecular weight of PLA, and the weight percentage of PLA in the solvent (10–20%). According to a previous report, polymer microneedles having higher molecular weight exhibit greater mechanical strength, as one might expect [23].

We observed that, with a higher concentration, the higher molecular weight of PLA resulted in higher viscosity. Excessively viscous polymer solution makes it difficult to fill the micron sized-PDMS molds, which leads to failure of the micro molding. Figure 1b shows our observations when the microneedle structure is fabricated under various conditions, and Figure 1c showed the mechanical strength of some leading candidates. As mentioned in the method part, the mechanical strength of the microneedle array was measured at 200 μm of strain. It was observed that microneedle arrays were not successfully fabricated when using acetone as dissolving solvent. This was due to excessive bubbles generated during the drying process. In the case of dimethylformamide, low viscosity and low surface tension of the PDMS surface were observed. These hindered the sophisticated micro molding of the microneedle array by causing overflow of the PLA solution from the mold by external vibration or shock. Based on these observations, a solution of Resomer 207S in DMSO (15 wt%) was selected for further experimentation. In the scanning electron microscopy (SEM) image analysis, the successful fabrication of PLA microneedle of 250~500 μm height was observed (Figure 1d). DMSO is regarded as a non-toxic and biocompatible solvent at low concentrations, and has been used in drug formulations for that reason [24]. In contrast, dimethylformamide (DMF) is a potent liver toxin that may cause serious health issues including abdominal pain, weakness, dizziness, and alcohol intolerance [25]. We expect that this form of microneedle fabrication based on DMSO-casting will be applicable to the sophisticated and versatile design of microneedles, including for the arrow head or undercut versions, in the future [26].

In addition, we observed how much DMSO solvent remains during the drying process (Figure 1e). After 330 min, ~98% of the DMSO was evaporated. This observation excludes the possibility of harmful effect from residual DMSO in the PLA microneedles. Considering that only a small portion of the microneedle tips of a microneedle array penetrates the skin, it can be seen that the amount of residual DMSO (which could potentially infiltrate the punctured skin) is negligible.

PLA has been widely used in 3D scaffolds in tissue engineering and implanted devices due to its biocompatibility and biodegradability. We examined how PLA microneedle arrays degrade or are hydrolyzed under near-physiological conditions (Figure 1f). The degradation of PLA was studied under various conditions. Proteinase K is known to catalyze the degradation of PLA effectively in previous studies was used for evaluation of biodegradability [27,28]. Interestingly, the higher the molecular percentage of PLA was in the casting solution, the higher degradation rate the PLA microneedle arrays had. After 23 days of incubation, the microneedle arrays made with 5%, 10%, and 15% casting solution showed the residual weight ratio of 91.39%, 82.72%, and 86.39%, respectively. Previous studies showed that the PLA concentration in a solution with organic solvent for preparing film or scaffolding affects the porosity and pore-size of the cavities in the PLA-based structure [29,30]. It was observed that porosity and pore size regulate the hydrolysis and degradation of PLA [31,32]. It seems that the differences in biodegradability in relation to the PLA concentration is due to differences in pore size or porosity in the structure. Further research on the structure of PLA scaffolds, especially of porosity during the drying process following solvent-casting seems to be necessary.

### 3.2. Mechanical Characteristics of the Microneedles

**Figure 2:**
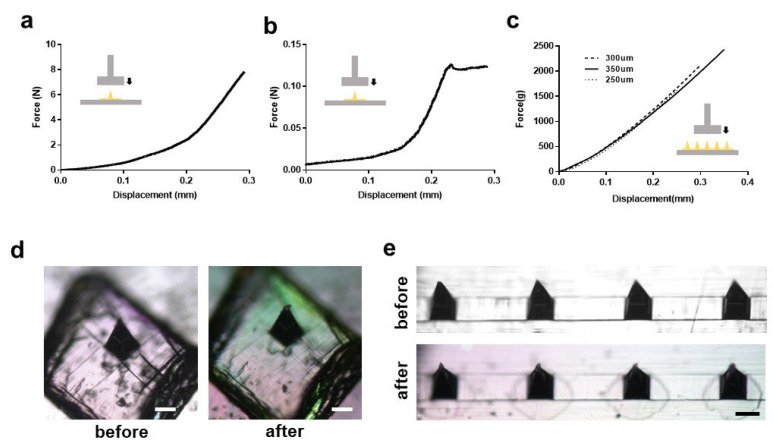
Mechanical characteristics of PLA microneedle. (**a**) Force-displacement curve of single PLA needle. Texture analyzer sensor moved axially at the test speed of 1.2 mm/s with trigger force of 0.003 N. (**b**) Force-displacement curve of single hyaluronic acid-based dissolving microneedle. Same analysis condition was applied. (**c**) Force-displacement curve of PLA microneedle array having various heights of 250, 300, and 350 μm. (**d**) Single needle compression test with 0.1 N. Scale bar, 125 μm. (**e**) Compression test of microneedle array with 5 N (0.06 N/needle). Scale bar, 125 μm.

To investigate whether the PLA microneedles fabricated using the solvent-casting method are appropriate for application to human skin, we measured the mechanical strength of the microneedle arrays. The results are shown as force-displacement curves in Figure 2a–c. A PLA microneedle array 300 μm high was tested using a texture analyzer. As shown in Figure 2a, fracture, or “needle failure” was not observed. Fracture refers to the point of drastic change in the force-displacement curve reflecting the practical morphological change of the structure [33]. This observation is consistent with previous studies of thermoplastic polymer microneedles. Some inflection points were observed in the graphs, but the curvature at these points (0.08 and 0.21 mm) was not significant. We fabricated hyaluronic acid-based dissolving microneedles based on our previous work, and analyzed their mechanical characteristics [18]. As opposed to the force-displacement curve of PLA microneedles, needle failure was observed with the dissolving microneedles (Figure 2b). In previous reports, it was observed that the minimum force required for a single microneedle to penetrate the skin is 0.058 N, which ensures that our PLA microneedles have enough mechanical strength to penetrate human skin [31]. 

In the texture analysis of the microneedle array, there was no significant difference between microneedle arrays with heights of 250, 300, or 350 μm (Figure 2c). 

In a compression test of a single PLA microneedle subjected to 0.1 N, the tip of the microneedle structure (~5% of total height) was slightly bent, but without significant deformation of the overall structure (Figure 2d). This observation is similar to the compression test of microneedle array with 5 N (0.06 N/needle) of compression force (Figure 2e). It is noteworthy that 0.058 N is necessary to penetrate skin, and that a PLA microneedle does not exhibit critical deformation at 0.058 N. 

### 3.3. Applications to Micro-Needling and Other Combined-Platforms

Microneedling, the process of repetitively puncturing the skin with a solid microneedle, has been studied in the past few decades. It has been utilized for the treatment of acne vulgaris, androgenetic alopecia, scars (atrophic acne, hypertrophic scars, or keloids), or melisma [34,35]. As indicated by reports on the processes of percutaneous collagen induction and reconstruction of epidermis or dermis mediated by microneedling it has also been used for skin rejuvenation [35]. This has resulted in various types of personal homecare devices, such as microneedle-rollers or stamps. A recent in-depth investigation on the molecular characterization of microneedling effects revealed that morphological changes in the skin are mediated by a number of processes. These include upregulation of genes associated with tissue remodeling and wound healing, epithelial proliferation and differentiation, immune cell recruitment, changes in heat-shock proteins, and downregulation of pro-inflammatory cytokines and antimicrobial peptides [36]. Advanced understanding of microneedling is expected to draw widespread attention in diverse fields in the future.

**Figure 3:**
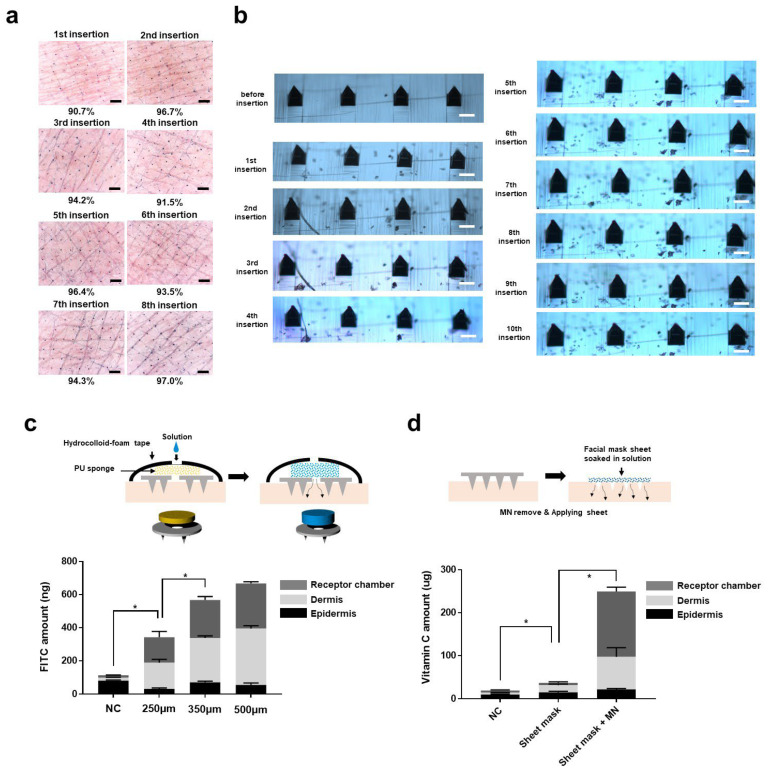
Applications micro-needling and other combined-platforms. (**a**) Penetration efficiency of PLA microneedle on the human skin. Same microneedle array was applied on the different sites of upper ventral forearm. After removal of microneedle from the skin, the pores generated on the stratum corenum were stained. Scale bar, 400 μm. (**b**) Optical microscopy image for PLA microneedle after each insertion during multiple-applications. Scale bar, 125 μm. (**c**) Applications of the PLA microneedle combined with PU reservoir-type patch. FITC solution (50,000 ng/mL) was injected on the PU sponge contained in hydrocolloid patch. After 16 h, the porcine skin, and the solution of Franz-cell reservoir were analyzed. (**d**) The PLA microneedle of which the length is 250 μm was applied the porcine skin. After removal of the microneedle array, the facial mask sheet soaked with the vitamin C 25% solution was applied on the needle-treated area. After 3 h, the vitamin C contents in skin substructures and Franz-cell reservoir was analyzed. The data represent the average of n  =  3 replicate experiments. Standard deviation bars are shown. * Significantly different (Student’s *t*-test, *p*  <  0.05).

We investigated whether the PLA microneedle structure would remain intact after multiple penetrations into human skin (Figure 3a). Skin penetration efficiency was measured when a microneedle array with height of 250 μm was used to penetrate human skin multiple times. The penetration efficiency was measured by dividing the number of stained punctured holes by the estimated number of holes that could be produced by the microneedles. It was observed that the penetration efficiency exceeds 90% in every application of microneedle until eight insertions of the same needle array (Figure 3a). There was slight flushing and edema after application of the PLA microneedles. However this condition disappeared within a few hours (data not shown). The severity and degree of symptoms was not different from an individual insertion during serial insertions.

After removing the microneedle array from the skin during each application, the microneedle structure was observed under an optical microscope (Figure 3b). The same area of the PLA microneedle array was observed each time. The tips of the microneedles (~5% of total height) were bent after the two insertions, but overall, the structure was intact without any fractures or crack as indicated in the previous single needle compression test in Figure 2a. It was observed that microneedle structure remained intact without any significant deformation after 10 insertions. Although PLA material is biodegradable, any PLA remaining could (rarely) cause a foreign body reaction and severe inflammation [32]. It can be concluded that our PLA microneedle exhibits appropriate mechanical strength and other characteristics, and does not leave behind any residual solid tips that could cause harmful effects, even after multiple applications of the same device. When considering the practical application of the PLA microneedles in various ways, not only a coating-based platform but also reservoir-mediated platforms were of interest. For this reason, we investigated whether our PLA microneedle could be applied in combination with a sponge (Polyurethane, PU) type reservoir and a sheet mask. We also considered how efficiently these platforms promoted transdermal delivery into porcine skin. 

First, we performed the Franz diffusion cell experiment after applying the sponge type reservoir combined with a PLA microneedle patch. Microneedles having heights of 250, 350, and 500 μm were used. Details of the experimental procedures are described in the methods Section 2. As expected, the application of microneedles promoted transdermal delivery of the FITC (as described in Figure 3c) by formation of micro-holes in the skin. Application of 250 μm PLA microneedles combined with PU foam showed a 3.3-fold increase of transdermal delivery of FITC. Compared to a negative control (topical application of FITC solution to the porcine skin), the amount of FITC delivered to the dermis and Franz cell reservoir was dramatically increased. This implies that the micro-pores and channels generated on the skin promote efficient delivery of drug molecules. It is noteworthy that a previous study showed that porcine skin has a stratum corneum (SC) that is 20–26 μm thick and an epidermis 30–140 μm thick [30]. The use of 350 and 500 μm PLA microneedle arrays both improved the transdermal delivery of FITC (5.6-fold and 6.6-fold, respectively). No significant differences in the delivery efficiency of 350 and 500 μm microneedles were observed. This observation implies that microneedles of greater length may not always result in higher transdermal delivery efficiency.

Previous reports have paid attention to the role of vitamin C in the skin. Vitamin C is known to be involved: (1) in collagen formation by acting as a co-factor for the proline and lysine hydroxylases; (2) as a potent antioxidant as a scavenger of free radicals; (3) in the inhibition of melanogenesis; and (4) in the differentiation or proliferation of skin component cells such as keratinocytes and fibroblasts [37]. Evidence about the variability and roles of vitamin C in intrinsic skin aging and extrinsic skin aging induced by ultraviolet radiation is still emerging [38]. For that reason, topical application of vitamin C in a cosmetic formulation has been proposed as an effective approach for protecting against intrinsic or UV-induced photoaging, while transdermal delivery of vitamin C has been challenging due to numerous factors. In this work, we tried to deliver vitamin C using a sheet mask soaked in a 25% solution. Previous studies showed that skin occlusion (covering of skin by tape, sheeting, or any impermeable dressing material) can increase transdermal delivery efficiency by increasing stratum corneum hydration, and possibly by altering the intracellular lipid organization [39]. The results of some research suggest that increase of the skin surface temperature and blood flow by skin occlusion could also affect the transdermal delivery efficiency. Sheet masks, also called “facial masks” or “mask-packs” in other cultural spheres, are widely used examples of one of the important categories in cosmetics and offer a skin occlusion effect. 

As in previous studies regarding the occlusive effect on transdermal delivery, the application of a sheet mask increased the delivery of vitamin C into skin 1.9-fold compared to application of a topical solution (Figure 3d). A dramatic increase (three-fold) of the vitamin C in the dermis was observed. Interestingly, application of a sheet mask and PLA microneedle (specifically, application of a sheet mask on porcine skin pretreated with a microneedle array) dramatically increased the transdermal delivery of vitamin C. There was a 12.9-fold and 6.8-fold increase of vitamin C delivery compared to that of the negative control (topical solution application) and sheet-mask-alone group, respectively. It is noteworthy that the amount of vitamin C delivered into the epidermis was not significantly different between the three groups, as if the epidermis was saturated. A similar result was also observed in the previous sponge-patch experiment. Previous studies performing Franz diffusion cell experiments indicate that the amounts of drugs (or target molecules) tend to become saturated in skin tissue, and some simulation also studies showed that the drug concentration in the epidermis reached a plateau within about 3 h [40,41]. 

### 3.4. Burst-Based Drug Release by Sugar-Containing PLA Microneedle Fabricated via Solvent Casting

As mentioned before, a solid microneedle system requires a drug-coating process on either the needle’s structure or the external drug supplying system. Here, we attempted to “load” the drug into the needle’s structure itself, with the intention that it would be released during the application of the needle to the user’s skin. For this purpose, we added to the PLA-DMSO solution and verified whether or not microneedles were fabricated. 

**Figure 4:**
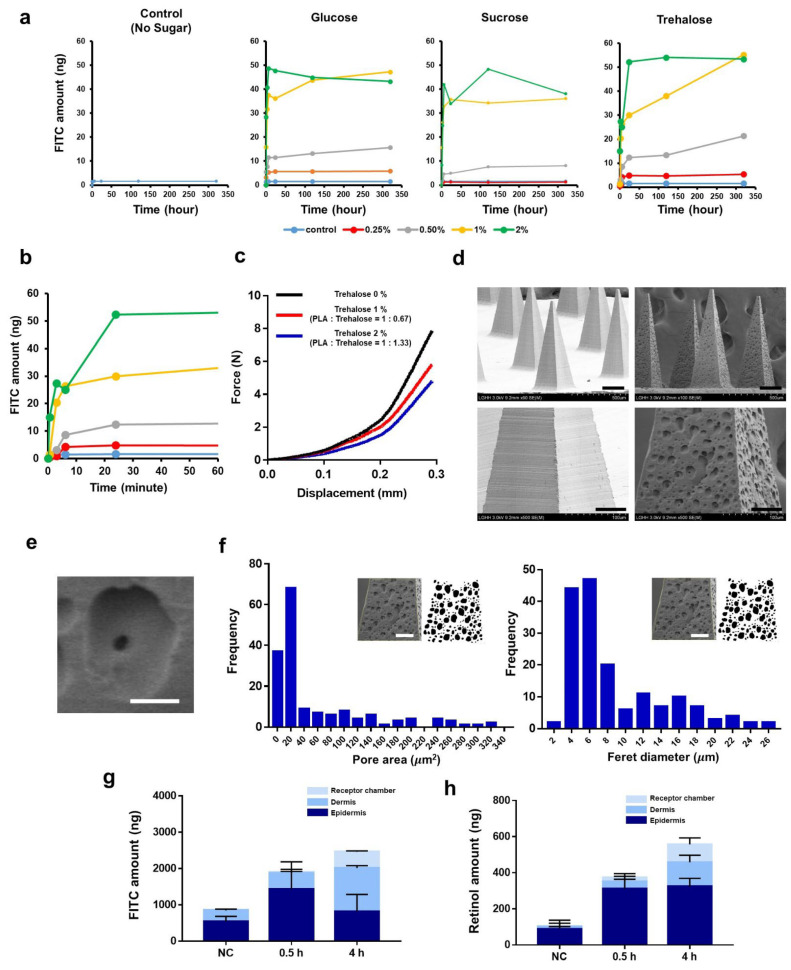
Burst drug-releasing PLA microneedle. (**a**) FITC-releasing pattern according to the kind of sugar and the composition of the casting solution (0.25–2%). (**b**) FITC-releasing pattern of trehalose-containing PLA microneedle (1% trehalose in casting solution; same data from panel (**a**)). (**c**) Force-displacement curves of single PLA needle arrays with different trehalose compositions. (**d**) SEM image for microneedle array containing trehalose. Scale bar for top row, 100 μm; Scale bar for lower row, 50 μm. (**e**) Pore formation inside outer pore (scale = 10 μm). (**f**) Characterization of surface pores; “Pore area” refers to two-dimensional area of pores on surface. Histograms show pore area (**left**) or Feret diameter (**right**). Scale bar, 50 μm. Transdermal delivery of FITC (**g**) and retinol (**h**) into porcine skin using burst drug-releasing PLA microneedle. Franz cell diffusion was performed, and target drugs were quantified using fluorescence spectrophotometer and HPLC.

We observed that three kinds of sugars were dissolved in the PLA/DMSO (15 wt%) solution within 2 wt%. At >2 wt% of sugar, we observed that sugar began to precipitate. Compared to sucrose and trehalose, glucose exhibited a more rapidly bursting drug release profile (Figure 4a). When the sugar content in the PLA/DMSO casting solution was low (0.25–0.5%), the release of FITC was limited. When the sugar content in the casting solution exceeded 0.5%, however, it was able to significantly release FITC. Sugar contents of 0.25, 0.5, 1, and 2% resulted in total matrix solid ratios (PLA:sugar) of 1:0.17, 1:0.33, 1:0.67, and 1:1.33, respectively.

As shown in Figure 4b (enlarged version of Figure 4a), most of the drug was released within 30 min, which enabled a “burst-drug release”. Interestingly, this rapid kinetics seems to be similar to those that have previously been obtained for dissolving microneedles [33,42]. 

Measuring the mechanical strength of the trehalose-containing PLA microneedle (Figure 4c) revealed that the strength decreased with increasing sugar content, but not significantly. Moreover, we did not observe any critical deformation or fractures.

Scanning electron microscopy (SEM) analysis revealed that the PLA matrix had a porous structure (Figure 4d). Based on a previous study into the relationship between porosity and drug release from a polymer matrix [43], we conclude that the formation of pores on the PLA matrix drove and accelerated drug release. Interestingly, we observed that smaller pores were generated inside these outer pores, which seemed to contribute to long-term sustained release under some specific conditions (Figure 4e). We did not observe this “pore in the pore” structure at samples prepared with lower sugar contents.

Next, we analyzed and characterized the pores on the surfaces of PLA microneedles (Figure 4f). The average pore area was 53.35

µm^2^ and the average Feret’s diameter (maximum) was 8.68 µm^2^. The two-dimensional porosity (pore area/surface area) was 33.9%.

Although the pore-forming ability of trehalose was similar to that obtained in a previous study [15], our approach exhibited a significantly different scale of release kinetics. In our research, trehalose achieved rapid drug release within 0.5 h, while in a previous study trehalose released the target drug from its PLGA matrix after 10 d [15]. The different pore sizes also seemed to affect the overall drug release kinetics (8.68 μm [our study] vs. <1 μm [ref. [15]]).

When comparing other similar studies for the rapid-release or delivery of drug cargo by rapid dissolution of a specific matrix, our drug-release system shows significantly more rapid kinetics (e.g., antibody delivery mediated by burst dilution of magnesium particles (~15 min for 50% of drug release) [44], including a PLGA/PLA matrix rapidly separated by bubble (~5 s for application, ~15 days for 50% of drug release from matrix) [16], exosome-loaded microneedles (~4 days for 50% of matrix dissolution) [13], and a programmed burst-drug release based on PLGA shell (30 min for application, ~10 days for burst drug release from matrix) [14]. To investigate how the target drug was released from the microneedle matrix during actual application to porcine skin, we loaded the model drugs, FITC, and retinol into 500 μm-high PLA microneedles. Briefly, we inserted the drug-loaded microneedles into porcine skin for 0.5 and 4 h, respectively, and then quantitatively analyzed the amount of each drug. 

Regarding the transdermal delivery of FITC, we observed that FITC was rapidly released from the microneedle and delivered into the skin within 1 h of application. The total amounts of FITC delivered for the negative control (NC) and after 0.5 and 4 h were 853.87, 1906.20, and 2641.36 ng, respectively (NC refers to the needless-PLA/trehalose/FITC matrix). The delivery of FITC for the NC group appears to have occurred through residual moisture on the skin surface dissolving the trehalose and enabling the FITC to effuse out. 

One of the distinguishing features was the molecular distribution between applications after 0.5 vs. 4 h. We observed decreased amount of FITC in the epidermis over time, suggesting that FITC diffused from the epidermis to the dermis and the reservoir as time passed. There was no significant difference in the total amount of FITC delivered between 0.5 and 4 h after application, implying that our PLA/sugar microneedle does not require more than 0.5 h of application time. 

Retinol, which is a lipophilic vitamin A derivative, has been widely used as a cosmetic ingredient to improve the appearance of skin by reducing fine lines and wrinkles. However, its poor water solubility and restricted transdermal delivery mean that specialized formulations are required. During the fabrication of our PLA microneedles, based on the DMSO-solvent casting process, we found that retinol was readily soluble in the DMSO-PLA-trehalose solution. We applied retinol-loaded PLA microneedles to porcine skin and characterized the transdermal delivery in the same manner as FITC. In total, we found that 413.7 and 506 ng of retinol were delivered 1 and 4 h after application, respectively. The needleless array (NC) delivered 102.4 ng of retinol in total. Due to its lipophilicity, we observed that less retinol was effused from the needle-lees array driven by residual moisture on the skin surface, in contrast to the delivery of FITC. Likewise, the amount of retinol in the dermis and reservoir increased with increasing application time, while its concentration in the epidermis did not change. In the delivery of retinol, the epidermis therefore seems to be a “rate-limiting step”, supporting the prior conclusion that the epidermis and dermis are only critical barriers for hydrophobic species [45].

Taking everything into account, our sugar-containing PLA microneedles exhibited the characteristics of both soluble and solid microneedles, including: (1) high mechanical strength of solid microneedles. As shown in Figure 2b (hyaluronic acid-based soluble microneedle) and Figure 4c, the prepared sugar-containing microneedles exhibited a similar mechanical strength to that of a PLA solid microneedle, with an approximately 45-fold higher stiffness than a soluble microneedle (slope = ∆F/∆displacement); (2) rapid drug release kinetics similar to soluble microneedles. Considering that soluble microneedles can have a wide range of dissolving times depending on the material (2 min–8 h) [46], our system requires a relatively short drug release time. Previous studies have highlighted that most soluble microneedles suffer from critical issues regarding physicochemical instability. Temperature and humidity can both significantly affect the mechanical characteristics of soluble microneedles during not only fabrications but also packaging and storage. Humidity is the most critical governing factor for mechanical stability due to the hygroscopic properties of said needles; low humidity results in the microneedle structure becoming more fragile, while high humidity results in a reduced mechanical strength due to them becoming “soggy”. For this reason, the extensive application of such microneedles in hot and humid countries still remains a hurdle, with high-stability packaging being investigated as a possible solution [47]. Our bursting drug-releasing PLA microneedles can overcome these practical issues regarding storage, packaging, and shipping, thereby increasing the applicability of microneedle platforms.

## 4. Conclusions

Microneedles have emerged as an appealing technology for effective transdermal delivery by filling the gap between topical applications and traumatic hypodermic needle injection. In particular, the demand for solid microneedles able to penetrate the stratum corneum effectively and create pores in the skin tissue is also increasing in the pharmaceutical and cosmetic industries. Here, we describe an effective approach to the fabrication of drug-releasing PLA solid microneedles using a solvent-casting method that enables a burst drug-releasing within 30 min. This method has advantages including great simplicity, broad geometric capability, and cost-effectiveness. Using high fidelity-replicas of microneedle arrays, this approach also exhibits good scalability, which is the most important virtue for mass-production in actual industries. It was verified that microneedle arrays having various lengths of needles (250–500 μm) could be fabricated with adequate mechanical characteristics. It was also shown that multiple applications of a microneedle array on human skin is feasible without any critical deformation or cracking of the needle structure. With respect to drug-releasing kinetics, our system exhibited quite rapid drug release of both hydrophilic and hydrophobic drug, which is comparable to a soluble microneedle. For this reason, our system possesses advantages for both solid microneedles and soluble microneedles, strong mechanical strength, and efficient drug delivery in limited application time. These advantages will help to overcome current issues regarding packaging or shipment due to the susceptibility to external force or humidity and also to improve the patient compliance.

As a promising fabrication approach compatible with mass-production, our PLA microneedle system will not only contribute to microneedle science, but also allow microneedles to get closer to eventual commercialization.

## Data Availability

The data that support the findings of this study are available from the corresponding author on request.
